# Ureido‐Ionic Liquid Mediated Conductive Hydrogel: Superior Integrated Properties for Advanced Biosensing Applications

**DOI:** 10.1002/advs.202401869

**Published:** 2024-07-03

**Authors:** Ruiying Ji, Shaopeng Yan, Zhiyu Zhu, Yaping Wang, Dan He, Kaikai Wang, Daofeng Zhou, Qike Jia, Xiuxiu Wang, Botao Zhang, Changcheng Shi, Ting Xu, Rong Wang, Rui Wang, Yang Zhou

**Affiliations:** ^1^ Cixi Biomedical Research Institute Wenzhou Medical University Ningbo 315300 China; ^2^ Laboratory of Advanced Theranostic Materials and Technology, Ningbo Institute of Materials Technology and Engineering Chinese Academy of Sciences Ningbo 315300 China; ^3^ Ningbo Cixi Institute of Biomedical Engineering Ningbo 315300 China; ^4^ Chemistry and Biomedicine Innovation Center (ChemBIC), State Key Laboratory of Coordination Chemistry, School of Chemistry and Chemical Engineering Nanjing University Nanjing 210023 China; ^5^ Pingshan Translational Medicine Center Shenzhen Bay Laboratory Shenzhen 518118 China

**Keywords:** biosensing, electrocardiograph monitoring, electrophoretic patches, integrated properties, ureido‐ionic liquid

## Abstract

Ionic conductive hydrogels (ICHs) have recently gained prominence in biosensing, indicating their potential to redefine future biomedical applications. However, the integration of these hydrogels into sensor technologies and their long‐term efficacy in practical applications pose substantial challenges, including a synergy of features, such as mechanical adaptability, conductive sensitivity, self‐adhesion, self‐regeneration, and microbial resistance. To address these challenges, this study introduces a novel hydrogel system using an imidazolium salt with a ureido backbone (UL) as the primary monomer. Fabricated via a straightforward one‐pot copolymerization process that includes betaine sulfonate methacrylate (SBMA) and acrylamide (AM), the hydrogel demonstrates multifunctional properties. The innovation of this hydrogel is attributed to its robust mechanical attributes, outstanding strain responsiveness, effective water retention, and advanced self‐regenerative and healing capabilities, which collectively lead to its superior performance in various applications. Moreover, this hydrogel  exhibited broad‐spectrum antibacterial activity. Its potential for biomechanical monitoring, especially in tandem with contact and noncontact electrocardiogram (ECG) devices, represents a noteworthy advancement in precise real‐time cardiac monitoring in clinical environments. In addition, the conductive properties of the hydrogel make it an ideal substrate for electrophoretic patches aimed at treating infected wounds and consequently enhancing the healing process.

## Graphical abstract

This study unveils a novel ionic conductive hydrogel, synthesized through a one‐pot copolymerization of ureido imidazolium ionic liquid, betaine sulfonate methacrylate, and acrylamide. Demonstrating multifunctional attributes, the ULAS hydrogel excels in mechanical adaptability, conductive sensitivity, self‐regeneration, and antimicrobial resistance. It shows potential in enhancing cardiac monitoring and wound healing applications, promising significant advancements in medical technology.

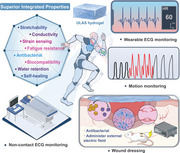

## 1 Introduction

Burgeoning bioelectronics has wide‐ranging applications in the detection and quantification of diverse physiological signals within the human body owing to its vital role in crucial cellular processes.^[^ ^1^, ^2^, ^3^, ^4^, ^5^ ^]^ These encompass a spectrum of physical indicators (strain, motion, and temperature),^[^ ^6^, ^7^, ^8^, ^9^ ^]^ electrophysiological measurements (electrocardiograms, electroencephalograms, and electromyograms),^[^ ^10^, ^11^, ^12^ ^]^ and a series of comprehensive profiles of biochemical information (pH, glucose levels, oxygen deficiency, enzymes, and an array of other vital biomolecules).^[^ ^13^, ^14^ ^]^ More recently, conductive hydrogels have attracted attention in the field of bioelectronics because of their unique combination of exceptional flexibility and high elasticity caused by their 3D soft structure and remarkable water‐retention properties.^[^ ^15^, ^16^ ^]^ Moreover, with the increasing demand for bioelectronic and human–machine interfaces, soft and flexible ionic conductive hydrogels have gained much interest over the past two decades owing to their intrinsic characteristics of high stretchability, transparency, tunable mechanical properties, consistent conductive phase, and biocompatibility.^[^ ^17^, ^18^, ^19^, ^20^ ^]^ The optical quality transparency is indispensable, affording the capacity to visually assess the internal state of both the devices and the underlying epidermis. Furthermore, the salience of ionically conductive hydrogels is underscored by the essential roles of ions and molecules in biological signal transmission.

Consequently, the investigation of ionically conductive hydrogels has garnered extensive scholarly attention. Jiang et al. fabricated a robust tough ionic hydrogel with exceptional ionic conductivity.^[^ ^21^ ^]^ The carboxyl‐functionalized, acryloyl‐terminated hyperbranched polycaprolactone was synthesized as the macro‐cross‐linker to impart an impressive range of stretchability (300–1100%) and marked toughness (14.1–44.1 MJ m^−3^) to the hydrogels. The conductivity attribute primarily arises from the incorporation of Fe^3+^ and Cl^−^ ions within the hydrogel. This demonstrates its promising potential for applications in the domain of flexible strain sensors. Such sensors can monitor a diverse array of human activities, ranging from joint movements to subtle facial expressions, in real‐time. Zhou et al. developed a groundbreaking polyelectrolyte hydrogel comprising acrylic acid (AAc), 1‐vinyl‐3‐butylimidazolium bromide (VBIMBr), and aluminum ions (Al^3+^).^[^ ^22^ ^]^ The incorporation of VBIMBr resulted in an exceptional ionic conductivity of 12.5 S m^−1^. In addition, the obtained hydrogels exhibited excellent self‐healing, anti‐freezing, and antibacterial properties. Polyelectrolyte hydrogel‐based sensors can consistently and repeatedly monitor human activity in a stable manner. Tan et al. introduced a straightforward one‐step methodology for synthesizing a resilient thermosensitive ion‐based hydrogel electronic device composed of poly(3‐dimethyl (methacryloyloxyethyl) ammonium propane sulfonate‐*co*‐AAc)/Al^3+^.^[^ ^23^ ^]^ This hydrogel device exhibited physical and chemical cross‐linking, resulting in excellent mechanical properties and high ionic conductivity. This hydrogel exhibited remarkable responsiveness and allowed multiple sensing processes, enabling the evaluation and monitoring of electrocardiograms comparable to those of commercial electrodes. By leveraging the precise temperature‐sensitive accuracy of these hydrogels, the simultaneous achievement of visualized qualitative observations for intelligent response, digitized measurement, and calibration of thermal stimuli becomes possible.

Although ionically conductive hydrogel bioelectronics are promising for diverse applications and have witnessed considerable recent advancements, they continue to face numerous challenges. Given the intricate nature of tissue‐device interfaces, bioelectronic applications require specific requirements and material properties.^[^ ^24^ ^]^ The foremost requirement is outstanding biocompatibility when exploiting these bioelectronics.^[^ ^25^ ^]^ Additional material features, for example, when conducting hydrogels are employed in electronic skin applications, it is imperative that these conductive hydrogels possess not only exceptional electrical conductivity but also exhibit notable stretchability, elevated mechanical strength, and resilience, thereby enabling them to withstand relatively substantial mechanical loads.^[^ ^26^, ^27^, ^28^ ^]^ Attached desirable attributes encompass interfacial adhesion and self‐healing capabilities, which enhance the practicality and durability of such bioelectronic systems.^[^ ^29^, ^30^, ^31^ ^]^ Beyond the aforementioned criteria, the incorporation of antibacterial properties is a pivotal aspect of implantable sensors.^[^ ^32^ ^]^ By preventing biofilm formation and preventing implant‐associated infections, antibacterial features significantly enhance the practical utility of these sensors.^[^ ^33^ ^]^ Within the domain of ionically conductive hydrogels, particularly those engaged in sensing applications necessitating direct skin interactions, an imperative yet frequently disregarded attribute of the surface as a prerequisite is the capacity for water retention. Imbuing hydrogels with moisture can cause electrode corrosion upon direct contact with their metallic counterparts, and evoke a sensation of humidity when interfaced with the human dermis. Consequently, these variables significantly compromise the comfort and functionality of sensing applications within a human physiological milieu.^[^ ^34^ ^]^ Moreover, the seepage of water and subsequent potential for ion leakage when an ionically conductive hydrogel interfaces with the skin present an inherent risk, potentially culminating in allergic reactions or post‐contact infections.^[^ ^35^ ^]^ In addition to poor water retention, a lack of self‐regeneration after dehydration is a major challenge for ionically conductive hydrogels.^[^ ^36^ ^]^ Not to the susceptibility of hydrogel matrices to moisture loss under mechanical pressure, these hydrogels are prone to rapid desiccation, often within a few hours of exposure to ambient environmental conditions. To curtail this propensity for moisture depletion, researchers have suggested using an elastomeric coating as a physical barrier for hydrogels. This strategy, however, introduces a layer of complexity in the manufacturing process, significantly amplifying the intricacy of hydrogel synthesis.^[^ ^37^, ^38^ ^]^ Such an increase in complexity impedes the practical application and broader dissemination of hydrogels. The concurrent integration of multiple functional properties into an all‐encompassing hydrogel matrix to address the intricate demands of bioelectronics is a formidable undertaking.

**Scheme 1:**
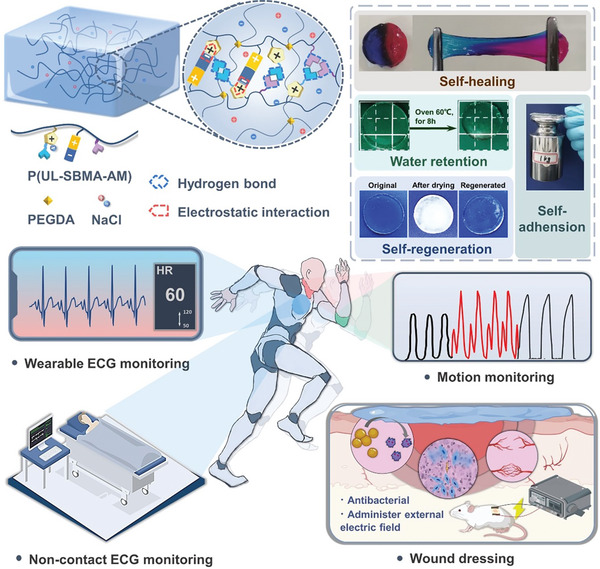
Schematic diagram of ULAS hydrogels and their applications for bioelectronic.

The ureido functional group represents one of the predominant and extensively employed reactive moieties in the realm of antimicrobial pharmaceuticals, which exhibits a unique capacity to establish multiple stable hydrogen bonds with proteins and receptor targets, thereby potentiating the antimicrobial response. Additionally, compelling evidence indicates that the incorporation of the ureido structure enhances the pharmacokinetic profile and ameliorates the inherent toxicity of pharmaceutical agents.^[^ ^39^, ^40^ ^]^ Similarly, ureido groups are commonly integrated into polymeric matrices, particularly in supramolecular gel systems. Owing to their formidable capacity for hydrogen bonding, hydrogels endowed with urea moieties are highly efficient units for hydrogen‐bonded assemblies within a consolidated supramolecular gel framework.^[^ ^41^ ^]^ Moreover, these ureido entities assume a dual functionality as both hydrogen bond acceptors and donors, representing integral noncovalent interactions within the gel matrix. This imparts the hydrogel with outstanding features, including promising toughness, resilience, self‐healing, and recyclability.^[^ ^42^, ^43^ ^]^ Ionic liquids (ILs), which are low‐melting‐point organic salts, exhibit distinctive physical, chemical, and biological characteristics, including elevated ionic conductivity, robust electrochemical stability, a broad electrochemical potential range, and broad‐spectrum antibacterial efficacy. Consequently, the integration of ILs into hydrogel matrices confers ionic conductivity to hydrogels, rendering them suitable for application in antibacterial sensing materials.^[^ ^44^, ^45^ ^]^ In this work, an ionic liquid imidazolium salt with a ureido backbone (UL) was synthesized and introduced into a hydrogel system (ULAS) together with betaine sulfonate methacrylate (SBMA) and acrylamide (AM) by facile one‐pot copolymerization. In the hydrogel matrix, the pervasive presence of two predominant non‐covalent interactions, namely hydrogen bonding and electrostatic interactions, endows hydrogels with superior mechanical properties, enhanced self‐healing, water retention, self‐regeneration attributes, self‐adhesion, and elevated conductivity. Owing to their exceptional conductive sensitivity, these hydrogels have extensive applications in tracking human biomechanics and are aptly suited for integration into both contact‐based and noncontact electrocardiography devices, facilitating precise and timely electrocardiogram recordings in practical clinical settings. Furthermore, the incorporation of urea‐based ionic liquids augments biocompatibility compared to commonly used *N*‐butylimidazolium ionic liquids and amplifies their antimicrobial efficacy. In conjunction with SBMA present in the hydrogel matrix, they collaboratively conferred a potent antimicrobial effect, effectively countering a wide spectrum of bacterial strains, including drug‐resistant *S. aureus*. Based on this premise, the application of this hydrogel as an electrotherapeutic patch augmented by advanced electrical stimulation substantially accelerates the healing process of critically infected wounds. This is attributable to the strategic molecular design and streamlined one‐pot synthesis method employed in the fabrication of this well‐designed ionic hydrogel system. The well‐designed ionic hydrogel ULAS is endowed with a constellation of desirable attributes, positioning it as a versatile, pliable, and durable wearable sensory device (**Scheme** **1**). Their potential is broad, ranging from cardiac surveillance in various clinical settings to expediting the recuperation of infected wounds, where they serve as electrotherapeutic patches.

## 2 Results and Discussion

### 2.1 Preparation and Mechanical Properties of ULAS Hydrogel

**Figure 1:**
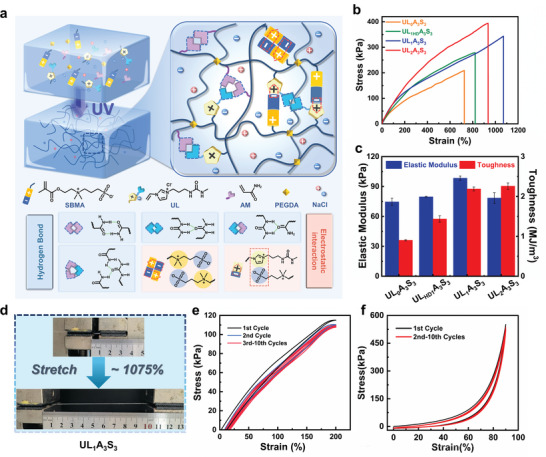
a) Schematic diagram of the preparation procedure of ULAS hydrogel. b) Tensile stress–strain curves of the UL_x_A_3_S_3_ hydrogels with different UL concentrations, and c) corresponding elastic modulus and toughness of UL_x_A_3_S_3_ hydrogels. d) Photographs of the UL_1_A_3_S_3_ hydrogel showing high stretchability. e) Stress–strain curves of UL_1_A_3_S_3_ in 10 successive loading–unloading cycles under the cyclic tensile strain of 200%. f) Stress–strain curves of UL_1_A_3_S_3_ with 10 cycles under the cyclic compressive strain of 90%.

The fabrication of the ureido‐based ionic liquid, which is a pivotal monomer within the ULAS hydrogel network, is described in the Experimental Section of the Supporting Information. Its molecular architecture was confirmed using proton nuclear magnetic resonance (^1^H NMR) and mass spectrometric analyses (Figure S1, Supporting Information). Subsequently, prototypical ULAS hydrogels were expeditiously and efficiently crafted through a singular step of stochastic polymerization involving the UL, AM, and SBMA. This synthesis was performed in an aqueous solution containing 1 m sodium chloride (NaCl), with polymerization induced by exposure to ultraviolet light for 10 min, as depicted in **Figure** **1**a. To systematically investigate the influence of the monomeric composition on the resultant hydrogel properties, the ULAS hydrogels were denoted as UL_x_A_y_S_z_. where x, y, and z quantitatively denote the molar concentrations of UL, AM, and SBMA, respectively, within the hydrogel matrix.

In our initial investigation, we scrutinized the implications of ionic liquids on the mechanical characteristics of hydrogels (Figure 1b,c). The hydrogel UL_0_A_3_S_3_, in the absence of ionic liquid incorporation, showcased a mere 726% elongation at break, along with a tensile strength of 209 kPa. Under identical conditions, the modulus and toughness of the UL_0_A_3_S_3_ were a mere 75 kPa and 0.9 MJ m^−3^, respectively. However, a significant increase in the stress of the hydrogels was evident as the concentration of the UL ionic liquid increased. With 1 m UL in the hydrogel system (UL_1_A_3_S_3_), there was an exponential growth in the elongation at the break of the hydrogel, surging to 1075%, and its fracture stress swelled to 343 kPa (Figure 1d). This significant mechanical reinforcement was primarily ascribed to intricate noncovalent interactions within the hydrogel matrix. This marked improvement in the mechanical integrity of the hydrogel was predominantly attributed to the intricate noncovalent interactions within the hydrogel matrix. Robust hydrogen bonding plays a pivotal role, particularly in the ureido group of the ionic liquid, the amide groups of acrylamide, and the interactions between these two groups. Additionally, the electrostatic interactions between the positively charged imidazole units and negatively charged sulfonate groups in the SBMA framework were critical contributors to the observed mechanical enhancements. In tandem with these investigations, it was observed that the incorporation of the UL substantially augmented both the elastic modulus and toughness of the hydrogel, which were quantified as 98 kPa and 2.19 MJ m^−3^, respectively. These enhancements indicate the significant reinforcement and toughening effects imparted by the UL ionic liquid. Further increasing the UL molar concentration to 2 m (UL_2_A_3_S_3_) increased the tensile strength of the hydrogel, reaching a peak at 394 kPa. In contrast, in comparison with UL_1_A_3_S_3_, a noticeable decrease in the elongation at break was observed, registering 936%, along with a marginal increase in toughness to 2.26 MJ m^−3^. This particular trend is attributable to an intensified network of hydrogen bonds and electrostatic interactions, which, while bolstering the tensile strength, concurrently imposes constraints on the flexibility of the hydrogel network, thus limiting further enhancements in strain. To further elucidate the influence of ureido incorporation into the ionic liquid on the mechanical properties of the hydrogels, a common ureido‐free ionic liquid (UL_HD_) was synthesized (see the Experimental Section and Figure S2, Supporting Information), and a comparative study was conducted with this UL_HD_ hydrogel, denoted as UL_HD1_A_3_S_3_. The UL_HD1_A_3_S_3_ hydrogel exhibited a markedly lower elongation at break (821%) and tensile strength (279 kPa) than UL_1_A_3_S_3_. These findings highlight the critical role of ureido hydrogen bonding in enhancing the tensile properties of hydrogels. Overall, the integration of ureido‐ILs into the hydrogel matrix was conclusively shown to significantly enhance the mechanical properties of the ULAS hydrogel.

The mechanical characteristics of the hydrogels were significantly influenced by the molar proportions of the other two monomers. Observations within a concentration range of 1–3 m for SBMA (from UL_1_A_3_S_1_ to UL_1_A_3_S_3_) revealed a significant increase in both the tensile strength of the hydrogel and its elongation at the point of rupture. Notably, the elongation at break increased from 198% to 1075%, concurrent with an increase in the tensile strength from a modest 160 kPa to a robust 343 kPa. Simultaneously, an increase in the SBMA molar concentration inversely affected the elastic modulus of the hydrogel, which registered a decline from 170 to 98 kPa. However, this is juxtaposed with a substantial surge in toughness, from 0.19 to 2.19 MJ m^−^^3^ (Figure S3a,b, Supporting Information). This marked improvement in mechanical properties is ascribed to the integration of SBMA, which instills electrostatic interactions within the gel matrix and serves as an efficacious energy dissipation mechanism. This strategic incorporation not only amplified the ductility and stretchability of the hydrogel but also mitigated the rigidity of the hydrogel network, thereby fostering an overall enhancement in the mechanical attributes of the hydrogels. Increasing the molar concentration of acrylamide from 2 to 3 m (UL_1_A_2_S_3_ to UL_1_A_3_S_3_) markedly enhanced the mechanical properties of the hydrogel network (Figure S3c,d, Supporting Information). This increase was manifested in the tensile strength, which escalated from 216 kPa to a notable value of 343 kPa, accompanied by an increase in the elastic modulus from 71 to 106 kPa. Concurrently, the toughness of the hydrogel experiences a significant rise, advancing from 1.31 to 2.18 MJ m^−3^, coupled with an extensional increase from 1008% to 1075%. However, it is noteworthy that a further increase in the acrylamide concentration to 4 m (UL_1_A_4_S_3_) while increasing the elastic modulus to 121.1 kPa, paradoxically resulted in a decrease in the fracture elongation to 845%. This was concomitant with a marginal decline in the tensile strength and toughness to 335 kPa and 1.74 MJ m^−3^, respectively. The observed reduction in the mechanical properties is predominantly ascribed to the unyielding architecture engendered by acrylamide polymerization, which establishes the structural backbone within the hydrogel matrix. Excess acrylamide catalyzes the profuse cross‐linking of the volumetric network. This hyper‐crosslinking jeopardizes the molecular integrity of the hydrogel, rendering it more prone to failure under tensile stress, thereby detrimentally affecting its mechanical attributes. This demonstrated the substantial influence of optimal acrylamide concentration balance on the comprehensive performance characteristics of the hydrogel. Consequently, we selected the UL_1_A_3_S_3_ hydrogel (ULAS) as the most suitable gel system for the next investigation due to its integrated exceptional tensile strength, strain at break, and toughness and the following scanning electron microscope (SEM) pictures revealed the 3D‐linked porous structure of the freeze‐dried ULAS hydrogel (Figure S4, Supporting Information).

In addition to their exceptional mechanical strength, the elastic recovery of hydrogels plays a pivotal role in extending their service lives and broadening their range of applications. To further elucidate this property, we conducted a series of successive cyclic tensile and compressive loading–unloading experiments. The results, as depicted in Figure 1e, from ten consecutive tensile loading–unloading trials at 200% strain, were instrumental in assessing the resilience of the ULAS hydrogel. Notably, the elastic recovery coefficients consistently exceeded 90% in each cycle (refer to Figure S5, Supporting Information), underscoring the pronounced elasticity and minimal hysteresis of the ULAS hydrogel. In addition, cyclic compressive tests, depicted in Figure 1f, show minimal deviation in the stress–strain curves across ten cycles under a strain of 90%. Crucially, the peak stress levels remain virtually unchanged. These observations collectively indicate that the ULAS hydrogel possesses impressive resilience, largely attributable to the synergistic integration of the physicochemical crosslinking processes. Such resilience and fatigue resistance underpin the long‐term sustainability and dependability of the ULAS hydrogels.

### 2.2 Sensing Ability and Durability of ULAS Hydrogel

**Figure 2:**
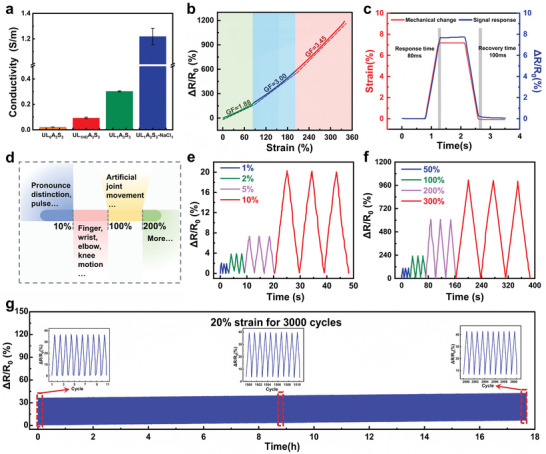
a) Ionic conductivity of the hydrogels. b) The linear fitting curve of relative resistance changes versus different strains. c) Response and recovery time of the ULAS hydrogel when used as a strain sensor. d) Illustration of correlation between the approximate range of strain scales and human activity. e) Resistance change rate responding to small tensile strains of the ULAS hydrogel. f) Resistance change rate responding to large tensile strains of the ULAS hydrogel. g) Resistance response of the ULAS hydrogel to continuous cyclic stretching of 20% strain beyond 3000 cycles.

In the present study, we examined the impact of UL on the ionic conductivity of hydrogel matrices. **Figure** **2**a shows that the integration of UL ionic liquids into hydrogels significantly augmented their conductivity, increasing from a baseline of 0.021 S m^−1^ (UL_0_A_3_S_3_) to an impressive 0.302 S m^−1^, ≈14.4‐fold increase. It is of particular interest to note that the conductivity exhibited by UL markedly outperformed that of conventional, unmodified ILs (UL_HD1_A_3_S_3_), which registered a conductivity of 0.093 S m^−1^. Our hypothesis is that this notable increase in conductivity can be primarily ascribed to the incorporation of polar functional groups via urea modification. These groups significantly bolster the intermolecular hydrogen bonding interactions within the hydrogel structure, thereby streamlining and enhancing ion mobility. Additionally, the UL ionic liquids exhibited excellent dispersion and compatibility within the hydrogel framework. This superiority is primarily evident from the higher solubility of urea‐based ionic liquids in water and their clearer and more uniform dispersion when mixed with AM and SBMA to form a prepolymer solution compared with that of UL_HD_. This harmonious integration is speculated to facilitate a more homogenous ion distribution and escalate the ion concentration, ultimately culminating in increased conductivity. In contrast, 1‐butyl‐3‐vinylimidazolium ionic liquid (UL_HD_), hindered by its inherently hydrophobic butyl chains, exhibits diminished ionic mobility within the hydrogel network, leading to a reduction in its conductivity. This study underscores the pivotal role of molecular modifications in tailoring the ionic properties of hydrogel systems and offers insights into the design of advanced materials with enhanced functional attributes. A further remarkable enhancement in the ionic conductivity of the ULAS hydrogels was achieved through the strategic alteration of the gelation solvent. By transitioning from pure water to a 1 m NaCl aqueous solution, the conductivity of the hydrogels increased to 1.216 S m^−1^. Significantly, the inclusion of NaCl substantially boosted the conductivity while concurrently preserving the mechanical properties of the ULAS hydrogels (Figure S6, Supporting Information). In light of these findings, all research pertaining to ULAS hydrogels was conducted in a 1 m NaCl aqueous solution, which ensured the dual attainment of superior ionic conductivity and preservation of the excellent mechanical properties of these hydrogels.

The ULAS hydrogel exhibited unique characteristics in terms of not only offering exceptional conductive properties and substantial strain sensitivity. Furthermore, this material highlights swift response and recovery attributes and is fortified with exceptional durability. Figure 2b illustrates the relationship between the mechanical strain and the relative change in electrical resistance (Δ*R*/*R*_0_) for the ULAS hydrogel. The gauge factor (GF), indicative of the strain sensor sensitivity, varies with the applied strain: registering at 1.88 for strains below 85%; increasing to 3.00 for strains between 85% and 200%; and further ascending to 3.45 beyond 200% strain threshold. Notably, the Δ*R*/*R*_0_ of the ULAS hydrogel demonstrates an almost linear augmentation from 0% to 350% tensile strain, evidencing its expansive sensing range. The dynamics of the ULAS hydrogel, specifically its response and recovery times, are critical metrics for its function as a strain sensor. These times were ascertained by deducing the lag from the electric response time during the loading–unloading cycle. As shown in Figure 2c, the hydrogel exhibited a response time of 80 ms and a recovery time of 100 ms under minimal strain conditions, positioning both metrics in the vicinity of 100 ms. This rapid action emphasizes the suitability of hydrogels for applications requiring quick and accurate sensing capabilities. Human body deformations exhibit a broad range of scales, extending from 0% to over 100%, as demonstrated in Figure 2d. Activities such as pronunciation and pauses are categorized as minor‐scale movements, typically comprising less than 10%. Conversely, movements involving the fingers, wrists, elbows, and knees were classified as major‐scale movements ranging from ≈10% to 100%. This distinction is crucial for our analysis of the multiscale sensing capabilities of the ULAS hydrogel sensor. As depicted in Figure 2e,f, the ULAS hydrogel sensor consistently demonstrates highly reversible and reproducible resistance changes (∆*R*/*R*_0_) across successive stretch/release cycles. These cycles were characterized by incrementally increasing strains, ranging from 1% to 10%, and further extending from 50% to 300%. These results unequivocally verify the robust and reliable multiscale sensing proficiency of the ULAS hydrogel sensor for human body monitoring applications. Furthermore, the durability of the ULAS hydrogel sensor's sensing capabilities was rigorously evaluated over 3000 uninterrupted loading–unloading cycles at a 20% strain, with a real‐time recording of ∆*R*/*R*_0_ signals. Figure 2g illustrates that the ∆*R*/*R*_0_ signals of the ULAS hydrogel sensor maintain exceptional stability and repeatability throughout the 3000 loading–unloading cycles.

### 2.3 Self‐Healing, Water Retention and Self‐Regeneration of the ULAS Hydrogel

**Figure 3:**
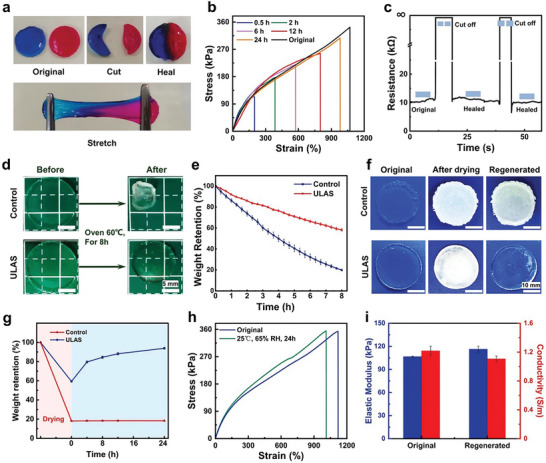
a) Photographs of the original, damaged, and healed ULAS hydrogels. b) Tensile stress–strain curves of the original and 0.5, 2, 6, 12, 24 h healed ULAS hydrogels previously cut into two halves. c) Real‐time resistance changes of the ULAS hydrogel during the cutting and healing process. d) Photographs of the control and the ULAS hydrogel under 60 °C in the oven for 8 h. e) Comparison of weight retention rate between the control and ULAS hydrogel under 60 °C environment. f) Photographs of the control and ULAS hydrogel original, after drying and regeneration at 20 °C and 65% RH. g) Weight retention rate of the control and ULAS hydrogel during the drying and regeneration period. h) Tensile stress–strain curves of the ULAS hydrogel before and after regeneration. i) Elastic modulus and conductivity of ULAS hydrogels before and after regeneration (20 °C, 65% RH) process.

The ULAS hydrogel network, imbued with a plethora of non‐covalent interactions, including intermolecular electrostatic interactions, hydrogen bonds, and dipole–dipole interactions, confers the exceptional self‐healing ability to the hydrogel. Incising the hydrogel with a scalpel and subsequently readhering the severed facets under minimal pressure significantly expedited the self‐healing process. Chromatic assays corroborated the robust self‐healing proficiency of the hydrogel; it maintained its excellent tensile strength and resilience after healing for 24 h (**Figure** **3**a). This attribute was further examined using tensile assays. As shown in Figure 3b, at a controlled temperature of 40 °C, the hydrogel exhibited a self‐healing efficacy of 52.5% over a period of 6 h, and this metric escalated to ≈90% after 24 h, thereby substantially reinstating its mechanical integrity. To substantiate the hydrogel's self‐healing proficiency in terms of ionic conductivity, resistance assays were performed before and after hydrogel rupture. The incorporation of the hydrogel into a real‐time resistive monitoring apparatus facilitated continuous observation of resistance fluctuations throughout the severing and subsequent healing phases. The hydrogel initially exhibited a resistance of ≈11 KΩ. Upon incision, the resistance abruptly increases to an infinite value. Remarkably, upon thorough readherence of the cleaved interfaces, the resistance of the hydrogel rapidly reverted to its foundational value, thereby indicating 100% efficacy in conductivity self‐restoration (Figure 3c). This dual process of both mechanical and ionic self‐healing substantially amplifies the utility within the domain of hydrogels in flexible electronic device applications.

The inherent propensity of hydrogel materials to lose moisture at their interfaces often results in suboptimal water retention. The frequent necessity of water application on these gels not only leads to an unwelcome sensation of dampness when in contact with human skin, but also accelerates corrosion in contact electrodes, thereby curtailing their applicability in biosensing technologies. Prior research has shown that glycerol effectively binds to water molecules through its hydrogen‐bonding interactions. This binding reduces the vapor pressure within the hydrogels, thereby mitigating the rate of moisture evaporation. In the current investigation, the integration of UL ionic liquids within the ULAS hydrogel matrix markedly enhanced its water retention properties. A comparative analysis was conducted using pristine acrylamide hydrogel as a control specimen. Both the control and the ULAS hydrogel samples were subjected to an environment of 60 °C, with periodic weight measurements taken at 20‐min intervals over 8 h, providing insights into their moisture retention efficacy under thermal stress. As shown in Figure 3d, a stark distinction was observed between the control group and ULAS hydrogel after an 8 h period. The control, a pure acrylamide hydrogel, experienced significant desiccation, maintaining only 19.8% of its initial mass and transforming into a substantially dried form. In contrast, the ULAS hydrogel preserved 58.2% of its original weight, effectively illustrating a substantially reduced dehydration rate relative to that of the acrylamide hydrogel, as highlighted in Figure 3e. The UL ionic liquid encapsulated within the ULAS hydrogel played an indispensable role in curbing water molecule evaporation and the ensuing loss. The urea groups embedded in the ionic liquid form an array of hydrogen bonds with water molecules, thereby considerably strengthening the affinity between the ionic liquid and water entities. Moreover, the exceedingly low vapor pressure of the ionic liquid primarily contributed to the exceptional water retention proficiency of the ULAS hydrogel.

The ULAS hydrogel, which is distinguished by its outstanding water retention capacity, is susceptible to dehydration under extremely arid conditions. Nevertheless, moderate restoration of the ambient temperature enables the freeze‐dried ULAS hydrogel to effectively reabsorb ambient water molecules, thus promoting its self‐regeneration. This phenomenon is depicted in Figure 3f,g, where the hydrogel, subsequent to lyophilization, markedly recovers its original characteristics within a 24‐h period at 20 °C and 65% relative humidity (RH), with the mass returning to over 94% of its initial value. This self‐regenerative feature is notably lacking in acrylamide hydrogel controls, which demonstrate minimal mass alteration following the drying process. Further investigations into the tensile characteristics of the ULAS hydrogel, following a 24‐h period of self‐regeneration (Figure 3h), indicated a significant recovery in the elongation at break to more than 90% of its original state, along with the maintenance of almost identical tensile strength. In addition, a modest increase in the elastic modulus from 107 to 114 kPa was observed (Figure 3i). Upon completion of the self‐regeneration process, the ULAS hydrogel underwent conductivity evaluation, revealing a marginal decrease from 1.218 to 1.106 S m^−1^ while preserving its superior conductive attributes (Figure 3i). This outcome underscores the dual regenerative capacity of ULAS, encompassing both mechanical and conductive properties. The commendable attributes of the ULAS hydrogel, encompassing self‐healing, water retention, and self‐regenerative capacities, not only provide it with distinctive advantages for prolonged use but also highlight its broad potential in diverse sectors such as biomedicine, sensory technology, and environmental engineering.

### 2.4 Self‐Adhesion of the ULAS Hydrogel

**Figure 4:**
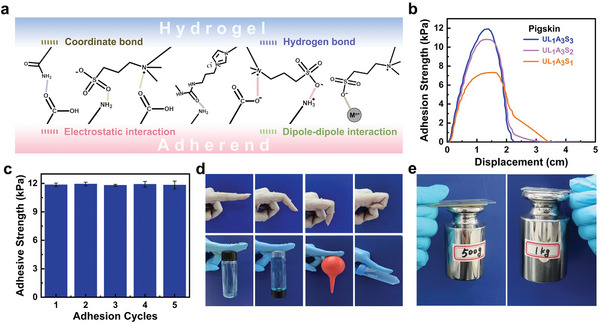
a) The self‐adhesion regulation mechanism of the ULAS hydrogel, respectively. b) Adhesion strength of the ULAS hydrogels with different SBMA concentrations to pig skin. The inset picture illustrates the corresponding testing method. c) Repeated adhesion of the ULAS hydrogel to pig skin. d) Adhesion of the ULAS hydrogel to various substrates (Fingers, PP, glass, rubber, and PE). e) Adhesion of the ULAS hydrogel to steel.

Electrostatic interactions, hydrogen bonding, and relatively weaker dipole–dipole interactions were conspicuously present at the interface between the ULAS hydrogels and their corresponding matrix substrates, endowing the hydrogels with exceptional self‐adhesive properties (**Figure** **4**a). To quantify the aptitude of these hydrogels for tissue adhesion, a lap shear adhesion test was performed on pig skin as a model tissue to evaluate their inherent adhesion properties. Illustrated in Figure 4b and acknowledging the critical role of SBMA in facilitating tissue adhesion, we embarked on a thorough study aimed at understanding the influence of varying SBMA concentrations on the firmness manifested by tissue adhesion. A directly proportional relationship was observed, where an increase in the SBMA composition was observed with a corresponding augmentation in adhesion strength. Using an optimal SBMA concentration of 3 m, the adhesion strength between the hydrogel and pig skin peaked at an impressive 11.9 kPa. This phenomenon was further examined through five iterative adhesion tests to confirm the consistent adhesion capability of the hydrogel (Figure 4c). Each iteration yielded adhesion strength values that exceeded 11.8 kPa, thereby confirming the resiliency and the lack of significant reduction across multiple tests. These findings underscore the strong adhesive properties of ULAS hydrogels for tissue adhesion in scientific and medical settings. Figure 4d shows the remarkable adhesive properties of the hydrogel across a diverse range of substrates including skin, steel, rubber, glass, and polypropylene (PP). A notable aspect of the hydrogel performance, as depicted in Figure 4e, is its remarkable adhesion to metallic surfaces. This was exemplified by the ability of the UL_1_A_3_S_3_ hydrogel to maintain robust adhesion under loads of weights measuring 500 and 1000 g. Significantly, the hydrogel demonstrated exceptional adhesive capabilities not only for tissues but also for metallic substrates. This dual adhesion attribute plays a pivotal role in its application as a flexible electrode material and sensor. By achieving a seamless interface with both the skin and circuitry, the hydrogel markedly reduced the interfacial impedance. This, in turn, enhances the sensitivity of the sensor and the signal‐to‐noise ratio, thereby bolstering its efficacy in these applications. The combination of adhesive versatility and ionic conductive properties posity makes this hydrogel a highly promising material for use in flexible electronics and sensing technologies.

### 2.5 In Vitro Antibacterial Properties and Biocompatibility of ULAS Hydrogel

**Figure 5:**
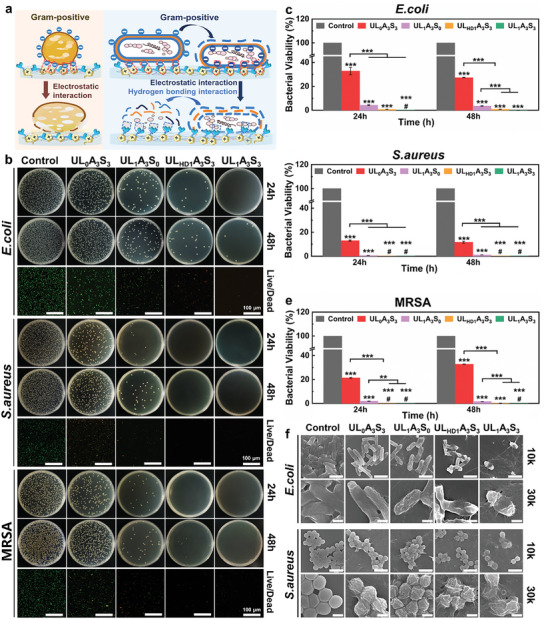
a) Mechanism of cell wall destruction from Gram‐negative bacteria and Gram‐positive bacteria by ULAS hydrogel. b) Photographs of *E. coli*, *S. aureus*, and MRSA colonies in agar plates after different treatments, and live (green)/dead (red) bacterial viability assay. Scale bar: 100 µm. Quantitative analysis of bacterial activities after treatments: c) *E. coli*, d) *S. aureus*, e) MRSA. # denotes that no bacterial colonies were observed on the agar plate. f) SEM images of *E. coli* and *S. aureus* after different treatments. Scale bar: 2 µm (10 000), 500 nm (30 000). Data were depicted as mean ± SD (*n* = 3), and one‐way ANOVA with Tukey's multiple comparison test was applied to analyze significant differences. *p < 0.05, **p < 0.01, ***p < 0.001.

In practical applications, it is critical to prevent bacterial proliferation on surfaces, particularly in wearable and implantable sensors, where maintaining functional integrity and averting infections are paramount. In the foundational phase of hydrogel monomer design, we gave precedence to the integration of the urea group, which is recognized for its potent antimicrobial properties. This study meticulously evaluated the hypothesis that incorporating urea moieties might amplify the antimicrobial properties of ionic liquids and probed the possibility of a synergistic antimicrobial interplay within the ULAS hydrogel system involving ionic liquids and SBMA (**Figure** **5**a). Our investigative approach encompassed the use of standard Gram‐negative *E. coli* and Gram‐positive *S. aureus* strains to gauge the antibacterial efficacy. Additionally, this study aimed to assess the antimicrobial activity of the hydrogels against Methicillin‐resistant *S. aureus* (MRSA), a notorious pathogen that presents formidable challenges in clinical therapy. This multifaceted bacterial analysis is pivotal in discerning the broader applicability and potency of hydrogel formulations for combating various bacterial threats. Using the polyacrylamide hydrogel as a negative control, bacterial cultures were incubated for 24 and 48 h to assess the antimicrobial potency of ULAS hydrogels via surface antibacterial assays, and the results are presented in Figure 5b. Remarkably, after incubation with the UL_1_A_3_S_3_ hydrogel, the agar medium did not contain bacterial colonies, indicating an eradication rate of ≈100% for all three bacterial strains. In contrast, the antimicrobial properties of the UL_0_A_3_S_3_ hydrogel, devoid of ionic liquids, were markedly inferior to those of both the hydrogen‐bond‐deficient ionic hydrogel variant UL_HD1_A_3_S_3_ and the hydrogen‐bonded ionic liquid‐infused UL_1_A_3_S_3_ hydrogels. Further investigation revealed that the UL_1_A_3_S_0_ hydrogel group retained the antimicrobial efficacy of UL. Comparative analyses highlighted that against *E. coli*, the UL_HD1_A_3_S_3_ hydrogel group exhibited reduced bactericidal efficiency compared to UL_1_A_3_S_3_; however, its effectiveness against *S. aureus* and MRSA remained unaltered. These observations were corroborated by meticulous quantitative assessments of bacterial viability (Figure 5c–e). The UL_HD1_A_3_S_3_ hydrogel exhibited antimicrobial activity against *E. coli*, curtailing bacterial survival to less than 1% at 24 and 48 h (0.7% and 0.8%, respectively). Notably, the UL_1_A_3_S_3_ hydrogel exhibited a significantly more pronounced inhibitory effect on *E. coli*, with bacterial survival rates approaching ≈0% compared to the control group. Consequently, the integration of the urea moiety into the ionic liquid matrix has been shown to augment the spectrum of antimicrobial activity while sustaining its inherent antimicrobial capabilities. This advancement has paved the way for the development of broad‐spectrum antimicrobial hydrogels with potential clinical applications. In addition to its capacity to inhibit bacterial adhesion, the quaternary ammonium moiety integral to the SBMA component within the ULAS hydrogel matrix disrupted bacterial membranes, thereby contributing to a synergistic antimicrobial mechanism. This phenomenon is substantiated by the documented antimicrobial efficacy of the UL_0_A_3_S_3_ hydrogel against Gram‐positive bacteria, particularly *S. aureus*.

To further elucidate the antimicrobial efficacy of the ULAS hydrogels, SEM was used to examine their effect on bacterial morphology. Figure 5c shows that in the control groups, *E. coli* and *S. aureus* retained smooth and unblemished surfaces. In contrast, in the UL_0_A_3_S_3_ and UL_1_A_3_S_0_ cohorts, noticeable wrinkling of the bacterial cell walls was observed, highlighting the influence of SBMA and UL ionic liquid on cellular wall integrity. Notably, in the UL_HD1_A_3_S_3_ and UL_1_A_3_S_3_ groups, pronounced wrinkling and occasional ruptures in the cell walls were evident, corroborating the superior antimicrobial functionality of the ULAS hydrogel as a consequence of the cooperative antibacterial mechanisms orchestrated by UL and SBMA. The extensive antimicrobial proficiency of the UL_1_A_3_S_3_ hydrogel is attributable to the confluence of the contributing elements. The quaternary ammonium motifs within SBMA are adept at compromising bacterial membranes, whereas the cationic segment of the ionic liquid disrupts the membrane functionality via electrostatic interactions. Additionally, the inclusion of ureido within the ionic liquid significantly bolsters its antimicrobial potency against *E. coli*, thereby enhancing the antibacterial activity of the hydrogels against Gram‐negative bacterial strains.

**Figure 6:**
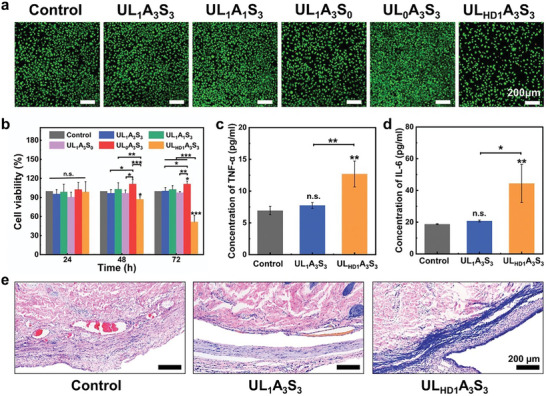
a) Live/dead staining of L929 cells after 72 h of incubation with the hydrogel extractions. b) CCK‐8 assay of L929 cells cultured in media treated with different hydrogel extracts for 24, 48, and 72 h. c) The serum concentration of TNF‐α after incubating with hydrogels for 7 days. d) The serum concentration of IL‐6 after incubating with hydrogels for 7 days. e) H&E staining of tissues surrounding the implants after 7 days. Data were analyzed using one‐way ANOVA followed by Tukey's multiple comparison test and presented as mean ± SD (*n* = 5 for b, *n* = 3 for c and d). n.s. indicated not significant, *p < 0.05, **p < 0.01, ***p < 0.001.

Substrate biocompatibility is imperative in the domain of wearable sensors, which require intimate contact with the human epidermis. An in vitro cytotoxicity assay was performed to evaluate the biocompatibility of the hydrogels. Following a 72‐h incubation period, during which viable cells emitted green fluorescence and nonviable cells red, it was observed that the control group and the UL_1_A_3_S_3_, UL_0_A_3_S3, UL_1_A_1_S_3_, and UL_1_A_3_S_0_ groups predominantly consisted of cells exhibiting normal spindle morphology with minimal occurrence of dead cells, with the notable exception of the UL_HD1_A_3_S_3_ group (**Figure** **6**a). Subsequently, the Cell Counting Kit‐8 (CCK‐8) assay was used to further assess cytotoxicity across different groups. Upon comparative analysis with a control group, the hydrogel variants UL_1_A_3_S_3_, UL_0_A_3_S_3_, UL_1_A_1_S_3_, and UL_1_A_3_S_0_ exhibited commendable cell viability, maintaining survival rates in excess of 90% over a span of three days without any substantial cytotoxic effects (Figure 6b). In contrast, the UL_HD1_A_3_S_3_ hydrogel group exhibited a surprising trend. Post 48‐h mark, the cell viability experienced a precipitous decline, cascading from 98% to 87%. This reduction was even more significant, plummeting to 51% after 72 h. This observation underscores the intricate and sometimes unpredictable nature of biomaterial interactions with the cellular environment, highlighting the necessity for comprehensive biocompatibility assessments in the development of materials for biomedical applications. In the field of ionic liquid technology, *N*‐butyl imidazolium ionic liquids, distinguished by their tetramethylene chains, are commonly used. However, our investigation revealed a critical aspect: at a concentration of 1 m in hydrogels, UL_HD_ not only manifested pronounced antimicrobial efficacy, but also significant cytotoxicity, as evidenced by a few academic sources.^[^ ^46^ ^]^

The in vivo biocompatibility of the hydrogels was assessed using a rat model of subcutaneous implantation. Skin tissues in contact with the UL_1_A_3_S_3_ and UL_HD1_A_3_S_3_ hydrogels, along with serum samples, were collected seven days post‐implantation. The sham group was used as the control group. Subsequent analysis revealed that the UL_1_A_3_S_3_ hydrogel implantation did not significantly elevate serum levels of TNF‐α and IL‐6 (refer to Figure 6c,d). Hematoxylin and eosin (H&E) staining, illustrated in Figure 6e, demonstrated minimal inflammatory cell aggregation at the tissue–hydrogel interface after seven days, indicating a subdued inflammatory response relative to the control. Conversely, the UL_HD1_A_3_S_3_ hydrogel, due to its cytotoxic effects, markedly increased serum TNF‐α and IL‐6 levels, as depicted in Figure 6c,d. This was corroborated by the pronounced increase in inflammatory cells in the H&E‐stained tissue sections in contact with the UL_HD1_A_3_S_3_ hydrogel (Figure 6e). These findings emphasize the necessity for in‐depth research on the biocompatibility of biosensing hydrogels. Our biocompatibility results indicate that a meticulous molecular architecture, especially the integration of urea‐based motifs into the ionic liquid framework, effectively reduces the cytotoxic nature of these liquids, thereby augmenting the biocompatibility of hydrogel matrices. This not only substantiates the viability of ULAS gels for biological applications but also heralds the innovation of biosafe monomers. Our strategy adopts a design philosophy reminiscent of sourcing structural concepts from pharmaceutical compounds, representing a strategic pivot in materials science toward biosafety and bioactivity. Consequently, this integration of distinctive properties qualifies the ULAS hydrogel as an exemplary material for pioneering bioelectronics applications.

### 2.6 Physiological and Bio‐Electronic Signals Recording with the ULAS Hydrogel

**Figure 7:**
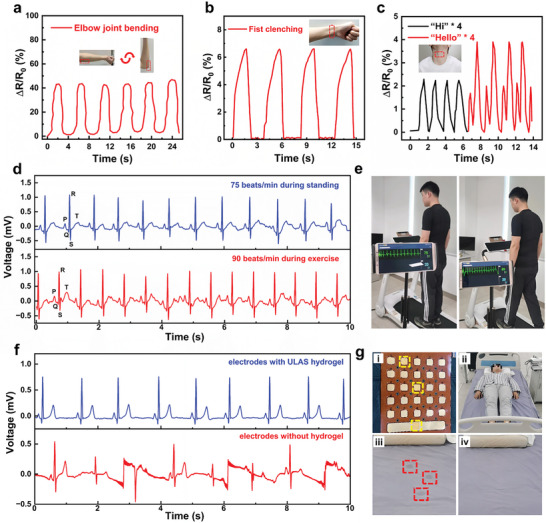
The recorded sensing response of the ULAS hydrogel sensor for a) elbow joint bending, b) fist clenching, and c) pronouncing “Hi” and “Hello”. d) ECG signals detected by the electrodes with ULAS hydrogel during standing and exercising. e) Diagram of monitoring the ECG signals during standing and exercising. f) ECG signals detected by the multilayered composite electrodes with and without ULAS hydrogel in sleeping heart monitoring. g) The device and scene of experiments. i) The photograph of the MLCE. ii) The scene of experiments. iii) The sheets with wet marks after using commercial conductive paste (Nihon Kohden, Japan). iv) The dry sheets after using ULAS hydrogel.

The ULAS hydrogel is notable for its extraordinary extensibility, heightened sensitivity, robust resistance to fatigue, intrinsic self‐healing capacity, excellent water retention, autonomous regeneration, and distinct ionic conductive properties, thereby satisfying the rigorous specifications essential for the development of long‐lasting wearable sensors and flexible electronic devices. Furthermore, this hydrogel demonstrated minimal cytotoxicity and superior self‐adhesion, rendering it suitable for direct integration with human tissues, which is a critical feature for biosensing technologies aimed at tracking a range of bodily movements. To explore the applicability of the ULAS hydrogel as a wearable strain/pressure sensor, it was attached to various human body segments to monitor activity in real‐time. As illustrated in **Figure** **7**a), when affixed to the elbow, the sensor effectively captured the intricacies of elbow flexion. This flexion was converted into distinct and reproducible electrical signals, thereby validating the dependability of the sensor under substantial strain. This characteristic is vital for precise monitoring of rehabilitation exercises, including wrist flexion, elbow bending, and knee flexion. Figure 7b illustrates the sensor affixed to the wrist, which meticulously records muscular contractions linked to hand clenching. Each clenching action was distinctly registered, indicating the efficacy of the sensor in tracking fluctuations in muscle strength, a capability that is crucial for providing essential diagnostic data for rehabilitative exercises after sports injury reconstructive surgeries. In addition, Figure 7c highlights the ability of the hydrogel to electrically respond to subtle strains. When positioned on the throat, the hydrogel demonstrates its ability to detect and distinguish between different vocal sounds, such as “hi” and “hello,” in real‐time, thus underscoring its prospective utility in voice recognition systems.

Electrocardiogram (ECG) is a pivotal diagnostic tool for discerning patterns in cardiac electrical activity, thereby facilitating the identification of various cardiac anomalies, including rhythm disturbances, compromised coronary artery perfusion, and electrolyte imbalances. Building on seminal research on hydrogel‐based sensors, our team engineered an innovative contact‐wearable ECG monitoring system. The system operates by first capturing ECG signals through a multilayered composite electrode (MLCE), which is principally composed of four distinct layers: a hydrogel layer, an MWCNT/PDMS composite film layer, a silver layer, and an insulation layer (TPU), as depicted in Figure S7 (Supporting Information). Subsequently, these signals undergo a series of processing steps, including filtration, amplification, and analog‐to‐digital conversion (ADC) within the signal acquisition circuit. After processing, the signals are transmitted to the smartphone interface via energy‐efficient Bluetooth low‐energy (BLE) technology, enabling real‐time visualization of the ECG waveform and accurate heart rate monitoring. Owing to the exceptional adhesive attributes, toughness, and biocompatibility of the ULAS hydrogels, the encapsulated flexible sensing electrodes conveniently interfaced with the skin of the chest. This advantageous property significantly mitigates the contact impedance and motion‐induced artifacts, which in turn boosts the accuracy of the measurements obtained. This enhancement subsequently increases the efficacy of the post‐measurement data processing techniques. As observed in Figure 7d,e, when the subjects were outfitted with an ECG‐enabled T‐shirt, they presented high‐quality electrocardiographic signals and clearly identifiable PQRST waveforms on a smartphone, whether stationary or involved in physical activity. In addition, cardiac rhythms can be inferred from ECG recordings. In addition, Figure 7d shows that the heart rate, while static, clocks at 75 beats per minute in comparison to over 90 beats per minute during periods of physical exertion.

Additionally, in this study, we integrated the ULAS hydrogel into an innovative noncontact ECG monitoring system meticulously tailored for individuals subjected to prolonged bedridden conditions. This intricate sensor apparatus is characterized by an MLCE framework accompanied by state‐of‐the‐art signal‐acquisition circuitry. The MLCE is principally composed of four distinct layers: a hydrogel layer, a sensitivity‐enhanced layer, an insulation stratum, and a reference electrode. The signal acquisition circuit is intricately designed and features a filter amplifier circuit, drive‐right leg circuit (DRL), ADC, and data transmission circuit. The operational mechanism of capacitive eletrocardiogram (cECG) signal involves capacitive coupling from the human skin to the sensitive layer of the electrode, a process facilitated by both pajamas and the bedsheet. This coupling is governed by the capacitance principle. The comprehensive design and functional principles of the system are illustrated in Figure S8 (Supporting Information). Figure 7f shows that the cohort employing hydrogel‐adorned electrodes exhibited cECG waveforms with salient signal attributes, including prominently defined T‐waves, QRS complexes, and P‐waves while being markedly devoid of substantial noise artifacts. Conversely, in scenarios where the hydrogel is omitted from the electrodes, the concomitantly low dielectric constants of both the bedsheet and pajamas precipitate a notable decrease in the coupling capacitance among the human epidermis, sleepwear, bedding, and sensing electrodes. This decrease in the coupling capacitance critically hampers the procurement of pristine high‐fidelity cECG signals.

Upon meticulous examination, it was evident that the ULSA hydrogel markedly surpassed its commercial counterparts in the domain of noncontact cECG monitoring systems (Figure 7g). Conventional clinical hydrogels, which are prevalent on the market, have considerable deficiencies chiefly attributable to their suboptimal water retention properties. This inadequacy is profoundly manifested when patients are stationary on monitoring beds, and the moisture content within these standard hydrogels is readily extruded, resulting in the saturation of bed linen and the formation of conspicuous water markings (positions marked in red in Figure 7g(iii)). This immediate interaction with moisture not only harbors the potential to instigate corrosion in metal electrodes (marked in yellow in Figure 7g(i)) but, more alarmingly, the persistent dampness of the linens escalates the peril of decubitus ulcers in individuals subjected to extended periods of bed rest. Conversely, the ULSA hydrogel was distinguished by its superior capacity to conserve water and sustain a state of hydration for extended durations (Figure 7g(iv)). This characteristic not only ensures consistent ionic conductivity but also maintains the bed linens in a perpetually dry state. Consequently, the ULSA hydrogel exemplifies its paramount significance in medical surveillance, particularly in its efficacious and enduring utilization as a noncontact cardiac monitoring apparatus for patients confined to their beds.

### 2.7 Electrotherapeutic Wound Dressing with the ULAS Hydrogel Electrodes

**Figure 8:**
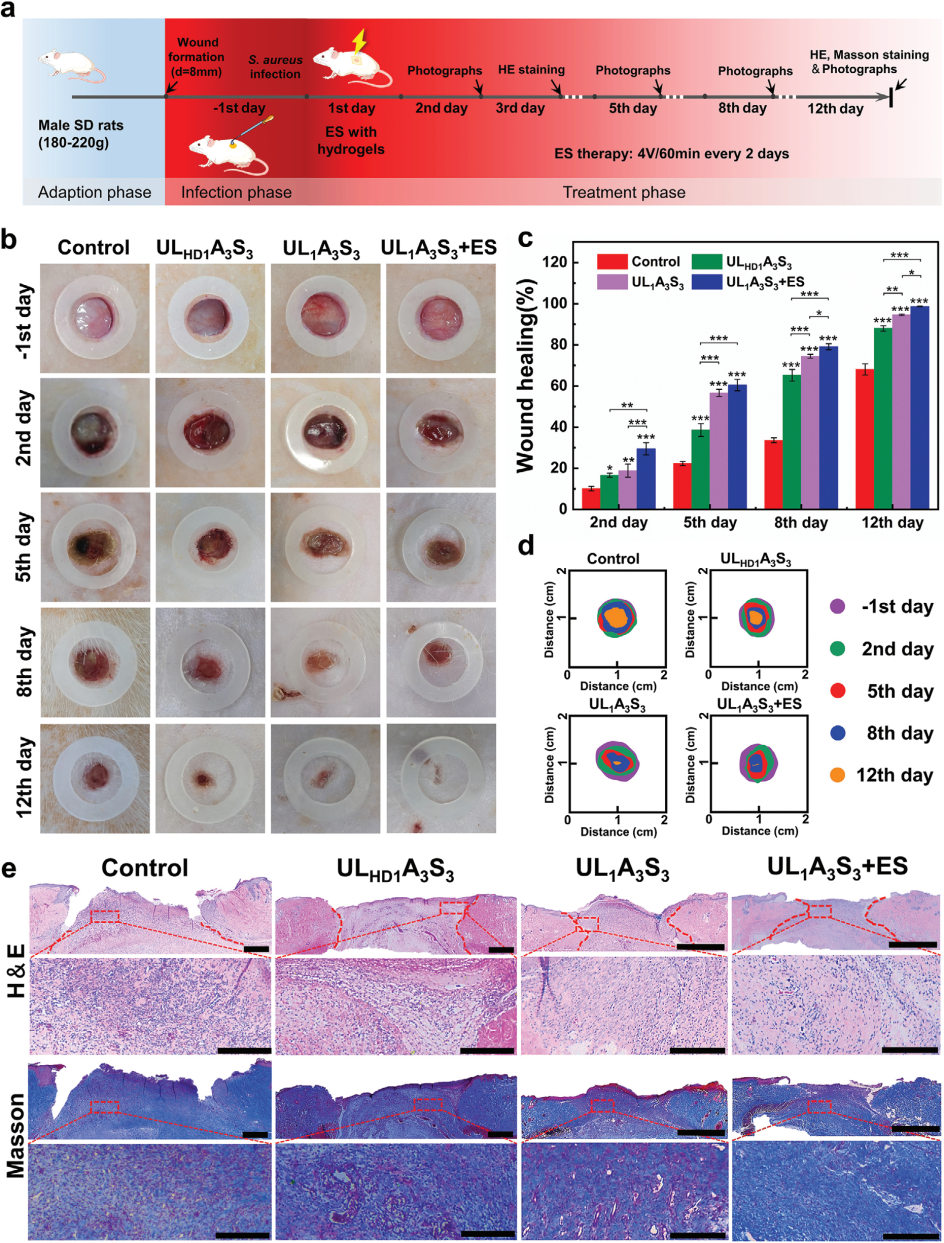
a) Schematic representation of in vivo wound healing experiment. b) Photographs of wounds on days −1, 2, 5, 8, and 12 from the different treated groups. c) Quantifying wound healing rates at different time points for each group. Data are depicted as mean ± SD (*n* = 3), and one‐way ANOVA with Tukey's multiple comparison tests was applied to the analysis of significant differences. *p < 0.05, **p < 0.01, and ***p < 0.001. d) Schematic diagram of wound healing under different treatments. e) Histopathological evaluations (H&E, and Masson staining) at the trauma site on the 12th day. Scale bar: 500 µm and 100 µm (zoom).

ULAS hydrogels exhibit remarkable flexibility, self‐adhesion, antibacterial properties, and biocompatibility, enabling optimal contact with injured tissues. This resulted in the formation of robust hydrogel–wound interfaces, rendering them ideal for use as electrotherapeutic patches owing to their inherent electroactive properties. To evaluate their efficacy, an *S. aureus*‐infected full‐thickness skin defect model was established in randomly selected SD rats to investigate the synergistic effect of the hydrogel in conjunction with electrical stimulation (ES) on infected wound healing. In this study, electroactive hydrogel dressings were integrated with a direct current (DC) power supply to administer an external electric field. ES intensities ranging from 0 to 500 mV mm^−1^ were applied to the UL_1_A_3_S_3_ hydrogel to determine the optimal wound‐healing rate (Figure S9, Supporting Information). The findings indicated a notable acceleration in wound recovery compared with that in the control group, with the most efficacious healing observed at an ES intensity of 400 mV mm^−1^. Beyond this threshold, the healing efficacy decreased to 500 mV mm^−1^. Consequently, an electric field strength of 400 mV mm^−1^ was selected for subsequent quantitative wound‐healing therapy. The commercial dressing Tegaderm film (3 M, USA) was used as a benchmark for the control group. The treatment methodology is illustrated in **Figure** **8**a. Two days after infection with *S. aureus*, a conspicuous infection was observed in the wounds of the control group. Conversely, groups treated with ionic liquids displayed a marked reduction in inflammatory responses, which substantially shortened the infection period, thereby facilitating wound healing. Histopathological evaluation on Day 2 H&E staining confirmed these findings. This analysis revealed minimal inflammation in the groups treated with UL_1_A_3_S_3_ and ES (Figure S10, Supporting Information). Notably, by Day 5, the control group continued to exhibit honey‐colored scabs, a hallmark of intense inflammation, whereas the other groups initiated the healing process. However, the group treated with UL_HD1_A_3_S_3_ gel encountered ongoing healing challenges attributable to the gel's suboptimal biocompatibility. In stark contrast, groups treated with UL_1_A_3_S_3_ and UL_1_A_3_S_3_ combined with ES demonstrated significantly improved healing outcomes by this juncture, as evidenced in the accompanying Figure 8b. On Day 12, the wound closure rate in the UL_1_A_3_S_3_ group was markedly higher (94%) than that in the control group (68%). The UL_1_A_3_S_3_+ES group achieved near‐complete wound healing with a closure rate of 98.6%, highlighting the pronounced synergistic effect of the UL_1_A_3_S_3_ hydrogel and ES in the wound healing process (Figure 8c). The comparative wound closure trajectories illustrated in Figure 8d further affirmed these results.

To comprehensively assess the impact of the UL_1_A_3_S_3_ hydrogel in conjunction with ES on actual wound healing, a detailed histological examination of granulation tissue across the wound bed was performed after a 12‐day treatment regimen. This examination utilized both H&E and Masson's staining techniques (Figure 8e). The results revealed pronounced disparities among the four study groups. Consistent with the wound closure rates depicted by hematoxylin and eosin staining, the wounds in the control group were in a continued state of repair, exhibiting noticeable inflammatory responses. Wounds treated with the UL_HD1_A_3_S_3_ hydrogel demonstrated less favorable healing outcomes than those treated with the UL_1_A_3_S_3_ hydrogel. Notably, wounds treated with the combined treatment of UL_1_A_3_S_3_+ES exhibited reduced granulation tissue width, along with enhanced connective tissue and epidermal structure relative to wounds treated solely with the UL_1_A_3_S_3_ hydrogel. Masson's trichrome staining was used to evaluate the collagen distribution within the granulation tissue. The role of collagen in skin regeneration, specifically in terms of its deposition and orientation, is crucial for wound repair. A homogeneous trend in collagen during the wound remodeling phase is indicative of enhanced wound healing efficacy. Notably, the UL_1_A_3_S_3_+ES group exhibited a pronounced presence of deposited and well‐oriented collagen structures that stained blue, indicating superior extracellular matrix remodeling and epithelial tissue reconstruction. In summary, these findings suggest that the electrical stimulation facilitated by the UL_1_A_3_S_3_ hydrogel augments wound healing. Consequently, it was postulated that the ULAS hydrogel possesses considerable electrotherapeutic potential, potentially serving as an electronic skin patch for the treatment of chronic wounds.

## 3 Conclusion

**Figure 9:**
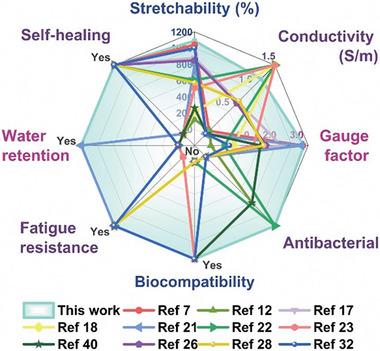
Comparison between ULAS hydrogel and previously reported hydrogels. (“Yes” denotes the presence of the function; “No” denotes the absence of or lack of corresponding data for the function).

In this study, we synthesized the UL and employed it as the principal monomer to engineer a novel hydrogel system through facile one‐pot copolymerization by incorporating SBMA and AM under UV exposure for 10 min. In contrast to the conventional UL_HD_, the UL significantly augmented the mechanical properties of the ULAS hydrogel. These properties include enhanced tensile strength, elongation at break, toughness, resilience, and fatigue resistance. The role of noncovalent interactions within the ULAS hydrogel matrix is critical. Specifically, the hydrogen bonds formed between the urea groups of UL and AM, along with the electrostatic interactions between UL and SBMA, are instrumental in boosting and maintaining the mechanical integrity of the gel. Moreover, when juxtaposed with UL_HD_, the presence of UL monomers markedly enhanced the conductive properties of the hydrogels. The ULAS hydrogel demonstrated superior conductivity and durability when subjected to the cooperative influence of UL and NaCl solutions. A particularly salient feature of the ULAS hydrogels is their extraordinary self‐healing, moisture retention, self‐regeneration, and self‐adhesion capabilities. Notably, moisture retention and self‐regeneration are relatively rare in conventional hydrogels. Moreover, the incorporation of UL monomers into the hydrogel matrix not only reduced the toxicity typically associated with ionic liquids but also significantly enhanced their antibacterial properties. When combined with SBMA, the ULAS hydrogel exhibited broad‐spectrum antibacterial efficacy, demonstrating robust activity against drug‐resistant *S. aureus* strains. This multifaceted functionality ensures the sustained effectiveness of the ULAS hydrogels in biosensing applications. Specifically, the potential of the ULAS for monitoring human biomechanics is noteworthy, particularly when integrated with both contact and noncontact ECG devices. This integration enables precise and real‐time ECG recordings in clinical environments, offering significant advancements in patient monitoring techniques. Additionally, the conductive properties of ULAS hydrogels make them ideal candidates for electrotherapy patches, particularly for accelerating the healing process of infected wounds. In summary, the innovative molecular engineering approach employed, characterized by the integration of urea functionalities within a vinyl imidazolium‐based ionic liquid framework, culminating in the development of ULAS hydrogels. This hydrogel is distinguished by its superior mechanical properties, enhanced strain responsiveness, water retention, and self‐regenerating abilities. These combined features collectively forge what is arguably the most robust and comprehensive performance spectrum observed in the realm of conductive hydrogels, as shown in **Figure** **9**. This novel strategy provides a transformative pathway for the synthesis of high‐performance hydrogels. Our research trajectory is set to broaden the scope of future ULAS hydrogel applications, particularly in the areas of biomedicine and medical technology. There is an ongoing commitment to diversify the array of high‐caliber hydrogel monomers to facilitate the production of a more extensive spectrum of advanced hydrogels.

## Conflict of Interest

The authors declare no conflict of interest.

## Author Contributions

R.J. conducted experiments and wrote the manuscript. S.Y., Z.Z., and Y.W. carried out material property experiments. K.W., D.Z., and Q.J. carried out sensing experiments. X.W. and B.Z. carried out animal and antibacterial experiments. C.S. and X.T. supervised the sensing experiments. R. W and R.W. contributed to the text revision. Y.Z. conceptualized and designed the study.

## Supporting information

Supporting Information

## Data Availability

The data that support the findings of this study are available from the corresponding author upon reasonable request.
