# Supplementary material for: Ureido‐Ionic Liquid Mediated Conductive Hydrogel: Superior Integrated Properties for Advanced Biosensing Applications
Source: Adv Sci (Weinh). 2024 Jul 3;11(33):2401869. doi: 10.1002/advs.202401869 (PMC11434023; doi:10.1002/advs.202401869)
Supplement: Supplementary file 1 — Supporting Information [file ADVS-11-2401869-s001.zip › advs202401869-sup-0001-SuppMat.docx]

**Supporting Information**

**Ureido-Ionic Liquid Mediated Conductive Hydrogels: Superior Integrated Properties for Advanced Biosensing Applications**

Ruiying Ji, Shaopeng Yan, Zhiyu Zhu, Yaping Wang, Dan He, Kaikai Wang, Daofeng Zhou, Qike Jia, Xiuxiu Wang, Botao Zhang, Changcheng Shi, Ting Xu, Jing Chen, Yang Zhou^*^

^a^ Cixi Biomedical Research Institute, Wenzhou Medical University, Ningbo, 315300, China

^b^ Institute of Biomedical Engineering, Ningbo Institute of Materials Technology and Engineering, Chinese Academy of Sciences, Ningbo, 315300, China

^c^ Ningbo Cixi Institute of Biomedical Engineering, Ningbo, 315300, China

^d^ Chemistry and Biomedicine Innovation Center (ChemBIC), State Key Laboratory of Coordination Chemistry, School of Chemistry and Chemical Engineering, Nanjing University, Nanjing, 210023, China

^*^ Corresponding authors

E-mail addresses: zhouyang876@nimte.ac.cn (Y. Zhou)

**Experimental section**

**Materials**:1-vinylimidazole (>99.0%), poly(ethylene glycol) diacrylate(PEGDA) and 2-hydroxy-2-methylpropiophenone (Photocure-1173) were obtained from Aladdin. 1-chlorobutane (>99.0%) and 3-chloropropyl isocyanate were obtained from Macklin. Methylamine (33 wt% solution in absolute ethanol) and NaCl were obtained from Sinopharm Chemical Reagent Co., Ltd. Cell Counting Kit-8 (CCK-8), dulbecco’s modified eagle medium (DMEM), fetal bovine serum (FBS) were obtained from Thermo Fisher Scientific. L929 cells were obtained from Shanghai Cell Bank. Luria-Bertani (LB) broth and agar were purchased from Beijing Soleibao Technology Co., Ltd. *S. aureus*, *E. coli*, and MRSA were obtained from the American Type Culture Collection (ATCC, USA).

**Preparation and characterizations of ionic liquids and hydrogels**

**Synthesis of** ***N*′-(3-Chloropropyl)-*N*′-Methylurea**: In a synthetic procedure conducted under an inert nitrogen atmosphere, methylamine (7.71 g) was combined with 3-chloropropyl isocyanate (10.0 g) in a 50 mL dichloromethane solution at 0 °C. This mixture was continuously stirred for 24 hours. Subsequently, the solvent was evaporated under reduced pressure. The resultant crude product underwent a purification process involving three washes with ethyl ether at 0 °C. After vacuum drying, a solid white product was isolated with a yield of 90%.

**Synthesis of UL**: N**′**-(3-chloropropyl)-N**′**-methylurea (11.5 g) and 1-vinylimidazole (7.9 g) were dissolved in 50 mL acetone. The resulting solution underwent stirring and refluxing at 70 °C for 24 hours. Following this, the solvent was evaporated under reduced pressure. The resulting crude product was subjected to three washes with ethyl ether. Subsequent vacuum drying at room temperature was performed. Further purification was achieved through silica gel column chromatography utilizing a trichloromethane/methane (10:1, v/v) eluent mixture. This procedure afforded the pure product, characterized as a clear viscous yellow liquid, with a yield of 70%.

**Synthesis of UL_HD_**: 1-chlorobutane (10.0 g) and 1-vinylimidazole (11.0 g) were combined in a 100 mL round-bottom flask. The mixture was stirred and refluxed at 70 °C for 12 hours. Subsequently, the reaction mixture was filtered and subjected to three thorough washes with ethyl ether. The product was dried under vacuum at ambient temperature, and further purification was conducted via silica gel column chromatography, employing a dichloromethane/methane (10:1, v/v) mixture as the eluent. This process yielded the target product as a clear viscous yellow liquid, with a yield of 67%.

**Preparation of ULAS Hydrogels**: The UL_x_A_y_S_z_ hydrogels with different UL/AM/SBMA monomer ratios were synthesized by a one-step free radical copolymerization process. The UL, AM, SBMA monomers, and NaCl were dissolved in deionized water for 10 min. Then, PEGDA (0.5 wt%) and 1173(0.5 mol% related to the monomer) were added and the obtained solution was stirred for 30 min until it completely dissolved, then exposed to 365 nm UV light for polymerization. The details of the recipe are presented in **Table S1**. ^1^H NMR data were acquired on a Bruker 400 MHz nuclear resonance spectrometer. The morphology and structure of the UL_1_A_3_S_3_ hydrogel were observed by Scanning Electron Microscope (SEM, S4800, Hitachi) after lyophilization.

**Table S1**. Recipe of precursor solutions for the synthesis of UL_x_A_y_S_z_ hydrogels.

|  | H_2_O | Monomer | | | Cross-linker | Initiator | NaCl |
| --- | --- | --- | --- | --- | --- | --- | --- |
|  |  | IL | AM | SBMA | PEGDA | 1173 |  |
| UL_1_A_3_S_3_ | 1.00g | 0.245g | 0.213g | 0.838g | 10μL | 6μL | 0.058g |
| UL_HD1_A_3_S_3_ | 1.00g | 0.245g | 0.213g | 0.838g |  |  | 0.058g |
| UL_0_A_3_S_3_ | 1.00g | 0 | 0.213g | 0.838g |  |  | 0.058g |
| UL_1_A_1_S_3_ | 1.00g | 0.245g | 0.071g | 0.838g |  |  | 0.058g |
| UL_1_A_3_S_1_ | 1.00g | 0.245g | 0.213g | 0.279g |  |  | 0.058g |

**Mechanical Testing Procedures:** A universal testing apparatus (Instron 5567) equipped with a 200 N sensor was employed for tensile evaluations. To mitigate dehydration of the hydrogel during these assessments, silicone oil was applied to its surface. The fracture toughness was quantified by integrating the area under the stress-strain curve using the device's proprietary software, while the modulus of elasticity was derived from the slope of the initial segment (5−20%) of the stress-strain curve. Additionally, compression tests were conducted. All experiments were performed at ambient temperature, and measurements were replicated five times using the same formulation; results are presented as mean ± standard deviation**.**

**Adhesive Performance Evaluation:** For lap shear assessments between pig skin and hydrogels, a universal testing device was utilized. Pig skin was prepared into strips measuring 30 mm in length and 8 mm in width, and was extensively rinsed with an ample volume of PBS buffer. Hydrogel samples, sized 8 mm × 14 mm × 1 mm, were sandwiched between two pieces of tissue, which were then pressed gently for 10 seconds prior to testing.

**Water retention tests**: The hydrogels were placed in the oven (60 ^o^C) for 8 hours. The water retention ability of hydrogels at different times was calculated by the following equation:

Water retention (%) = m_t_/m_0_ × 100%

where m_0_ and m_t_ were the weights of hydrogels at first and t, respectively.

**Self-regeneration tests**: The hydrogels were placed at ambient environments (25^o^C and 65% RH) for different times. The self-regeneration ability of hydrogels was determined by the following equation:

Weight retention (%) = m_t_/m_0_ × 100%

where m_0_ was the initial weight of undehydrated hydrogels, and m_t_ was the weight of dehydrated hydrogels after a self-regeneration period of t.

**Conductivity tests**: Four-probe resistivity tester (ST2258C, China) was employed to measure the resistivity of the hydrogel. The change in resistance of the hydrogel was recorded by using an electrochemical workstation (CHI600E, Chenhua, China). The resistance was determined using Ohm’s law (R = U/I) and expressing the relative shift in resistance due to diverse strains as GF = [(R−R_0_)/R_0_]/ε = (ΔR/R_0_)/ε, where R_0_ and R are the resistance of the original and stretched hydrogel, separately, and ε is the strain on the hydrogel. The detection of human motion signals was achieved by attaching the hydrogels directly to the human body.

**Antibacterial assay in vitro**: The sterilized hydrogels with a diameter of 6mm were immersed in LB medium with *Staphylococcus aureus* (*S. aureus*, 10^6^ CFU/ml), *Escherichia coli* (*E. coli*, 10^6^ CFU/ml), and Methicillin-resistant *Staphylococcus aureus* (MRSA, 10^6^ CFU/ml), respectively, and then incubated for 24h or 48h in a constant temperature incubator at 37^o^C. Then the hydrogels were sonicated in 500μL PBS buffer for 5 min to prepare the bacteria suspension. The bacterial suspensions were spread on a solid medium, the bacteria were incubated on LB medium at 37℃ for 24h, then the colonies were counted to quantify the bacterial viability. The bacterial viability rate was calculated as follows:

The bacterial viability rate (%) = C_x_ / C_control_ × 100%

where C_control_ and C_x_ were the colonies on the control and different sample groups (x=UL_0_A_3_S_3_, UL_1_A_3_S_0_, UL_HD1_A_3_S_3_, and UL_1_A_3_S_3_), respectively.

Additionally, the Live/Dead staining was recorded using a confocal microscope to confirm the bacterial viability of the bacterial suspensions cultured for 48 h. Simultaneously, the bacterial suspensions cultured for 24 h were transferred to silica wafers, fixed with 2.5% glutaraldehyde for 6 h at 4^o^C, dehydrated for 15 min in 50%, 70%, 90%, and 95% ethanol, and left for 30 min in 100% ethanol. The dried bacteria were coated with gold and used for SEM imaging.

**In Vitro Biocompatibility Assessment**: The biocompatibility of hydrogel extracts was evaluated using L929 murine fibroblasts. Initially, cells were cultured in a 6-well plate at a density of 50,000 cells per well for 24 hours. Subsequently, these cells were exposed to hydrogel extracts for 72 hours, followed by analysis using Live/Dead staining observed under a confocal microscope to assess cell viability and morphological integrity. Furthermore, cytotoxicity was quantitatively assessed using the CCK-8 colorimetric assay, a standard method to evaluate cytocompatibility. For this assay, L929 cells were seeded in 96-well plates and exposed to various hydrogel extracts. After co-incubation periods of 24, 48, and 72 hours, CCK-8 reagent was introduced to each well, and incubation continued for an additional 2 hours. Absorbance was measured at 450 nm to determine the viability of cells, with cell viability expressed relative to that of controls treated with DMEM culture medium (OD control).

**In Vivo Biocompatibility Evaluation**: Twelve rats weighing between 180-220 g were randomly assigned to three experimental groups. To evaluate the biological compatibility of UL_1_A_3_S_3_ and UL_HD1_A3S3 hydrogels in vivo, the hydrogel formulations were implanted into the dorsal region of the experimental animals. Untreated control rats received PBS buffer injections. Following a seven-day observation period, tissue samples from the implantation sites were excised for hematoxylin-eosin (H&E) staining to assess histopathological changes. Additionally, serum samples were collected to evaluate inflammatory responses, specifically TNF-α and IL-6 levels, using ELISA kits.

**In vivo wound healing**: A full-thickness wound rat models were created to assess the effect of the UL_1_A_3_S_3_ hydrogels with ES on wound repair. The rats were anesthetized with isoflurane and four full-thickness circular wounds of 8 mm in diameter were surgically created on the dorsal skin of every rat. In order to establish infection models, All the groups were treated with suspensions of S. aureus (100 μL, 1×10^8^ CFU/mL), and sterile gauze was applied. The 4 wounds on every rat were divided into 4 groups, including the control group (Tegaderm film dressing), the UL_1_A_3_S_3_ hydrogel group, the UL_1_A_3_S_3_ hydrogel with ES group, and the UL_HD1_A_3_S_3_ hydrogel group. The photographs of the skin defects in different groups were recorded by a digital camera on day 2, 5, 8 and 12, and the wound healing process was evaluated by monitoring the wound area. The wound area (%) was calculated from the equation below:

Wound healing (%) = [(A_0_− A_t_) / A_0_] × 100%

where A_0_ is the initial area and A_t_ is the wound area obtained on day n (n= 2, 5, 8, 12)
To assess the details of wound repair, the tissue samples were harvested and performed by H&E staining and Masson trichrome staining for histological analysis. All animal experiments were performed in accordance with National Institutes of Health animal care guidelines. Animal protocols were approved by the Institutional Animal Care and Use Committees on Animal Care (Nanjing University, approval No. BLARC-202110063).

**Statistical Analysis**: All tests were repeated three or more times and all results were presented as mean ± SD. ImageJ was used to obtain the quantitative results of the wound area. Statistical differences among the groups were assessed by one-way ANOVA followed by Tukey’s multiple comparison test using GraphPad Prism 9.0 and analyzed as follows: n.s., no significance; *, P < 0.05; **, P <0.01; ***, P < 0.001.

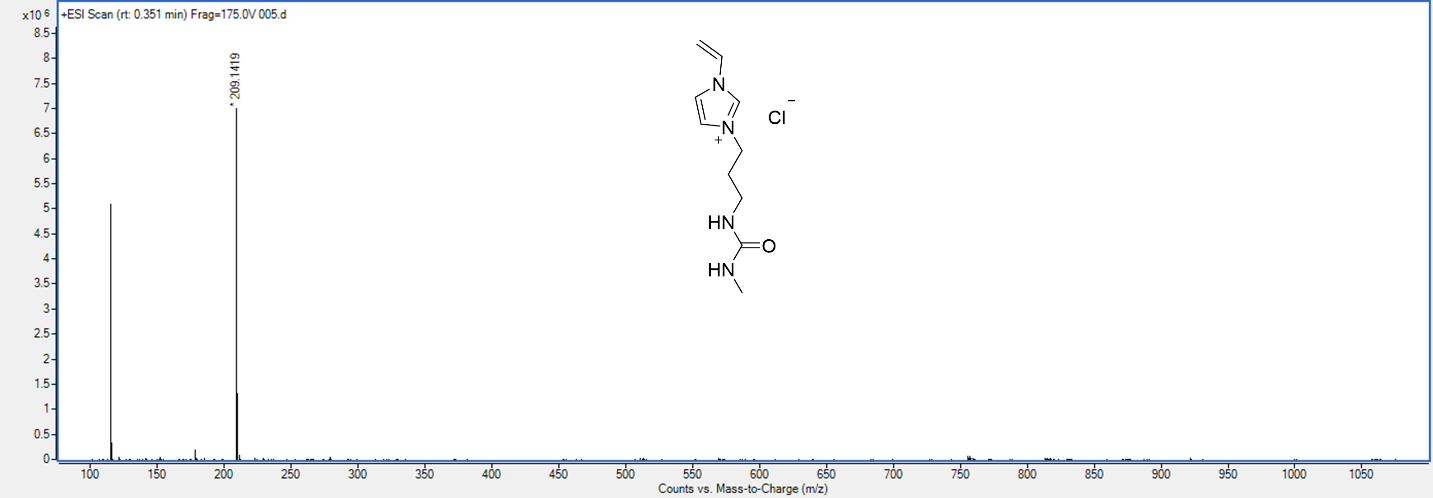


**Figure S1**. ^1^H NMR spectrum and mass spectrometry data for UL ionic liquids.

**Figure S2**. ^1^H NMR spectrum and mass spectrometry data for UL_HD_.


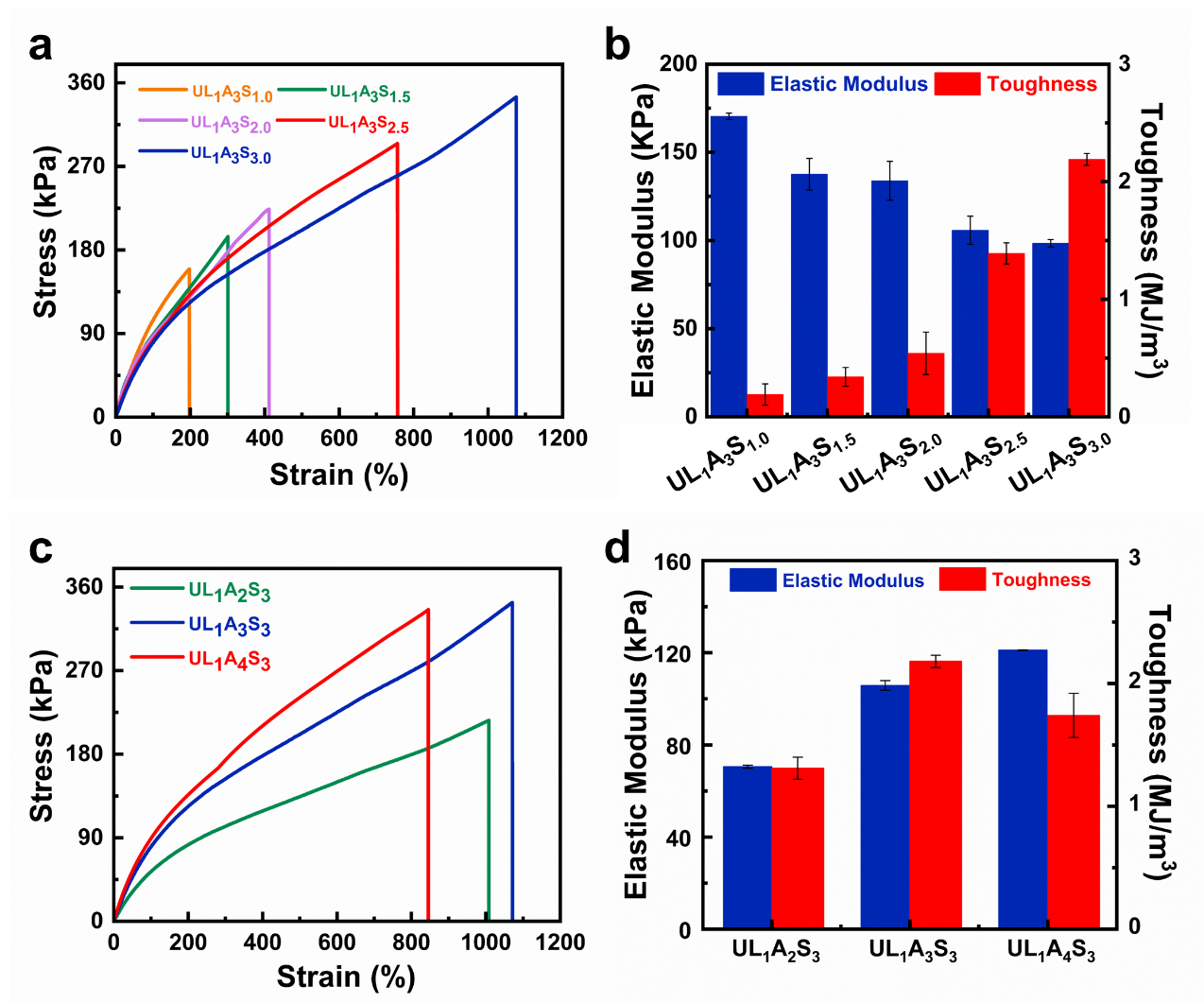


**Figure S3.** a) Tensile stress–strain curves of the UL_1_A_3_S_z_ hydrogels with different AM concentrations, and b) corresponding elastic modulus and toughness of UL_1_A_3_S_z_ hydrogels. c) Tensile stress–strain curves of the UL_1_A_y_S_3_ hydrogels with different SBMA concentrations, and d) corresponding elastic modulus and toughness of UL_1_A_y_S_3_ hydrogels.

**Figure S4**. SEM images of freeze-dried UL_1_A_3_S_3_ hydrogel.


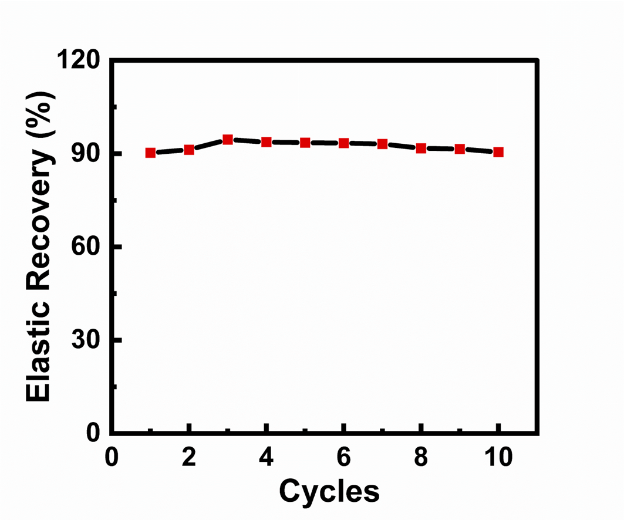


**Figure S5**. Elastic recovery of the ULAS hydrogel.


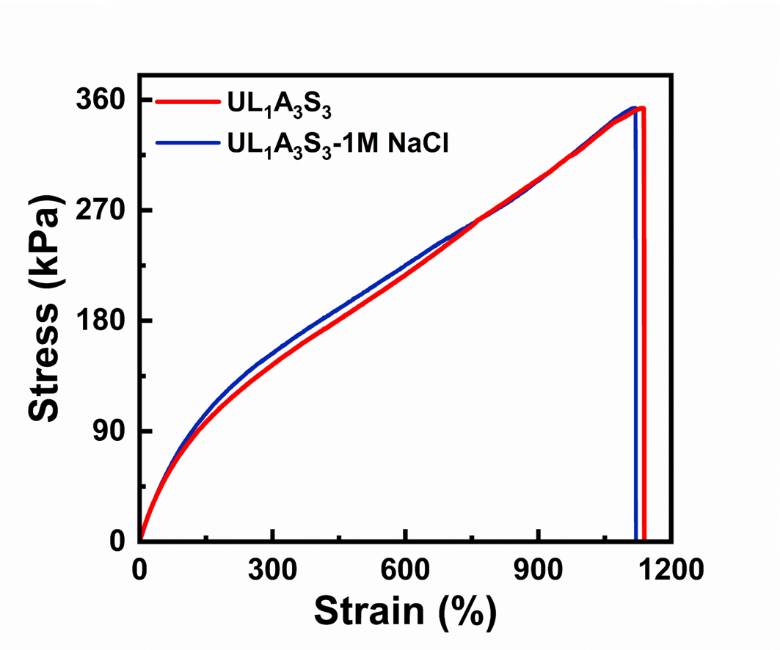


**Figure S6**. Tensile stress–strain curves of the ULAS hydrogels with different solvent (water or 1M NaCl).


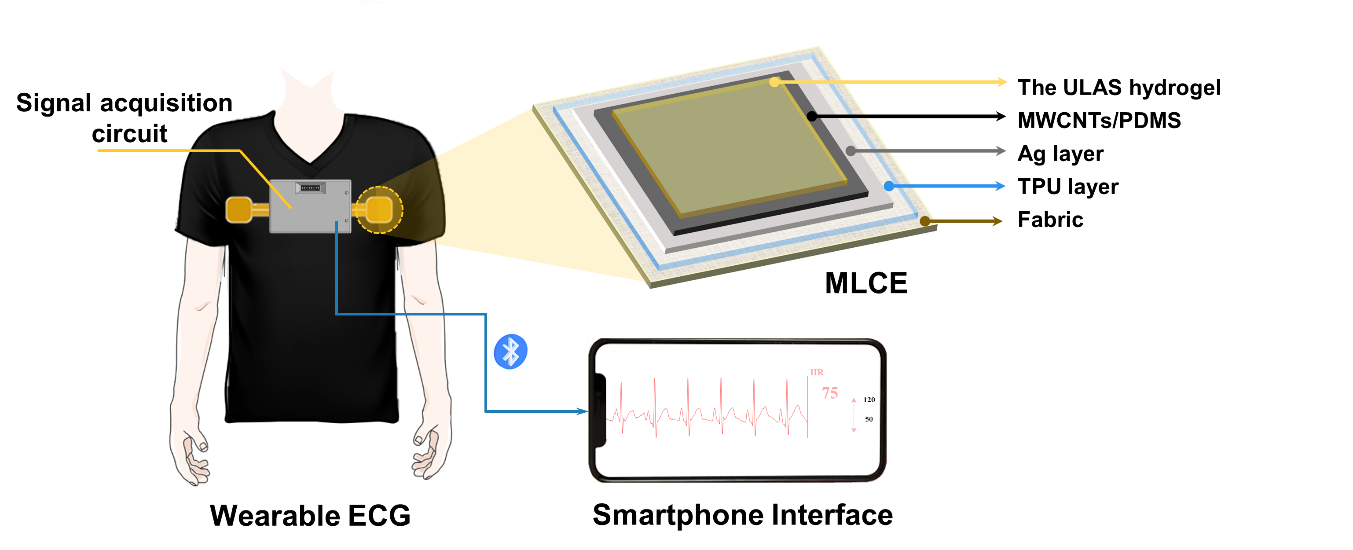


**Figure S7**. Diagram of the wearable ECG monitoring system, and configuration of MLCE used in the system.


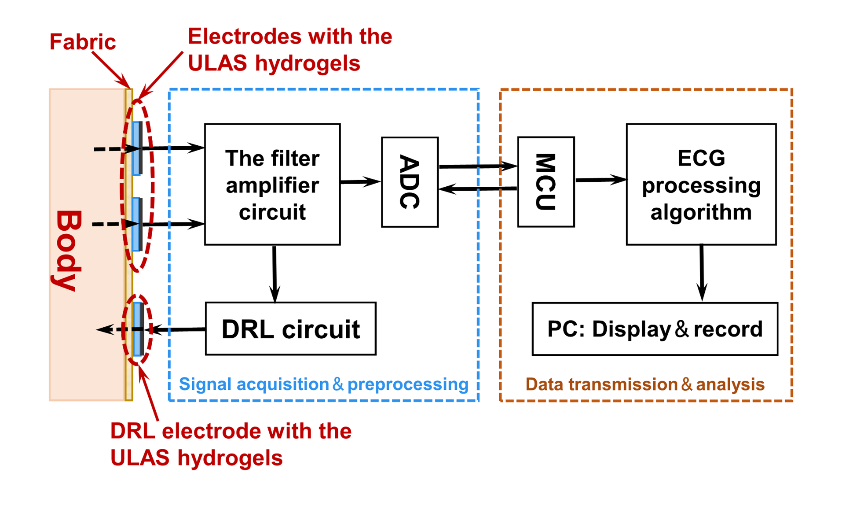


**Figure S8**. Diagram of system frame in non-contact cECG monitoring system. (ADC: analog to digital converter. MCU: microcontroller unit. DRL: drive right leg).





**Figure S9**. Quantifying wound healing rate applying the UL_1_A_3_S_3_ hydrogel with ES from 0-500 mV/mm.


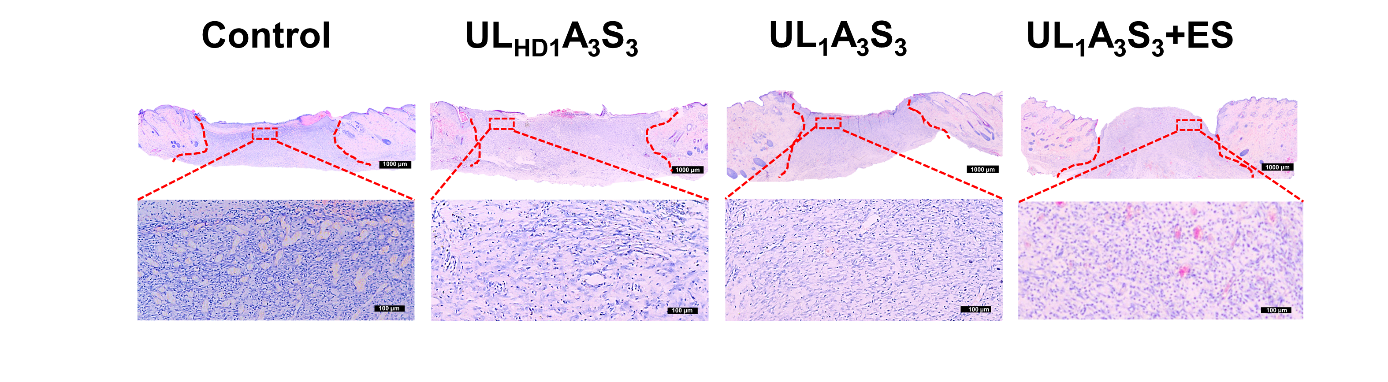


**Figure S10**. H&E staining at the trauma site on the 3rd day. Scale bar: 1000 μm and 100 μm (zoom).
